# Nephrotic syndrome with rectus sheath hematoma: a case report

**DOI:** 10.1186/s13256-024-04388-4

**Published:** 2024-03-10

**Authors:** Ai Fujii, Yuto Matsuda, Tomohisa Yabe, Hayashi Norifumi, Keiji Fujimoto, Masahide Yamazaki, Hitoshi Yokoyama, Kengo Furuichi

**Affiliations:** 1 Department of Nephrology, Kanazawa Medical University School of Medicine, 1-1 Daigaku, Uchinada, Kahoku, Ishikawa 920-0293 Japan; 2 Keiju Medical Center, Nanao, Japan

**Keywords:** Nephrotic syndrome, Abdominal pain, Rectus sheath hematoma

## Abstract

**Background:**

Rectus sheath hematoma is a rare presentation often associated with abdominal trauma and anticoagulant therapy. Here, we present a patient with severe rectus sheath hematoma accompanied by nephrotic syndrome who achieved significant clinical improvement without the need for invasive treatment.

**Case presentation:**

A 72-year-old Japanese woman was referred to our hospital for the treatment of nephrotic syndrome. She was receiving steroid and anticoagulant therapy. Then she had abdominal pain and she was diagnosed with spontaneous rectus sheath hematoma by abdominal computed tomography. She received transfusion and was managed conservatively with bed rest, which led to improvement in abdominal pain.

**Conclusion:**

Despite the absence of trauma history, rectus sheath hematoma should be considered in patients at risk of vascular failure, including those receiving anticoagulant or steroid therapy, those who are elderly, and those with nephrotic syndrome.

## Introduction

Rectus sheath hematoma, a serious presentation with high mortality, typically presents with sudden sharp, severe, and persistent pain. Risk factors for rectus sheath hematoma include chronic kidney disease and anticoagulant, steroid, and immunosuppressant therapies [1]. Rectus sheath hematoma should be considered in patients with abdominal pain, and abdominal computed tomography is necessary for definitive diagnosis. Established treatment protocols for rectus sheath hematoma are lacking [2].

Here, we present the case of a patient who was receiving steroid and anticoagulant therapy for nephrotic syndrome and developed rectus sheath hematoma, which improved with noninvasive treatment.

## Case presentation

A 72-year-old Japanese woman was referred to our hospital with hard pitting edema of lower extremities and increased weight. Her medical history included hypertension and nephrotic syndrome, which was in remission for three years.

Laboratory tests at admission revealed the following: leukocyte count, 8770/mm^3^; hemoglobin, 9.1 g/dL; hematocrit, 23.6%; serum creatinine, 0.73 mg/dL; blood urea nitrogen, 26.5 mg/dL; aspartate transferase, 21 IU; alanine transferase, 10 IU; creatine phosphokinase, 121 IU/L; and lactate dehydrogenase, 457 IU/L. Urinalysis showed 4 + proteinuria, and the selectivity index was 0.12. The complement levels such as C3, C4 and CH50 were not decreased. The coagulation factor was normal and immunological tests were negative. Serological tests for hepatitis B and hepatitis C were negative. She was diagnosed with the relapse of nephrotic syndrome and initiated steroid pulse treatment with methylprednisolone (500 mg for 3 days) with prednisolone (1 mg/kg/day) as post-therapy. Additionally, sustained heparin (10,000 U/day) was used to prevent thrombosis. Urinary protein and serum albumin levels gradually improved.

**Fig. 1:**
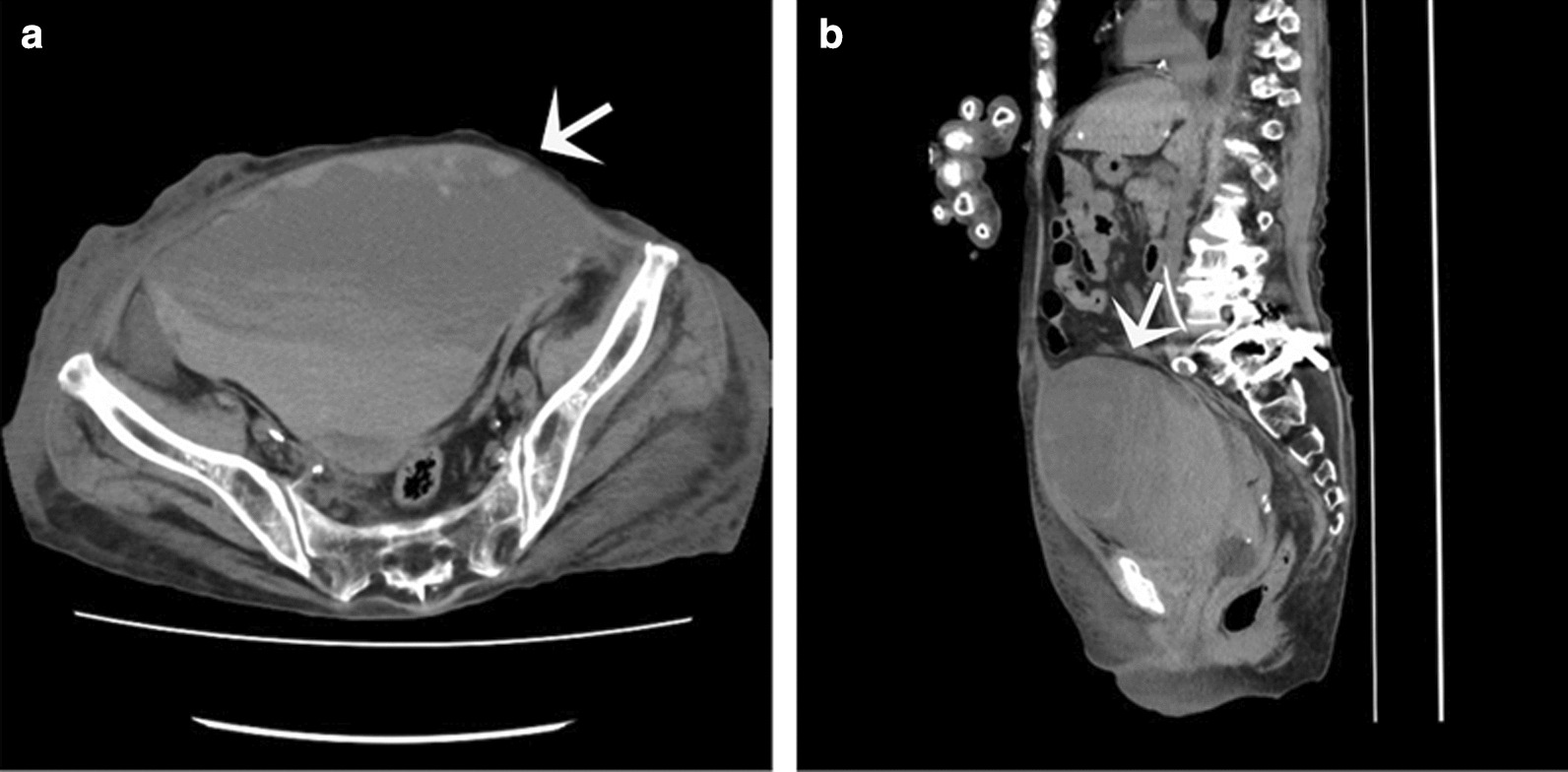
Non-contrast-enhanced abdominal computed tomography (CT) scan at caused abdominal pain. **a** Axial image. **b** Sagittal image. CT scan shows a hematoma in the left rectus sheath (arrow)

However, she developed edema affecting only the right lower limb seven days after treatment initiation. Her blood pressure was decreased, and she complained of abdominal pain. Her hemoglobin was 5.8 g/dL, and the leukocyte count was elevated at 13.580/mm^3^. Computed tomography confirmed marked asymmetry of the rectus sheath (Fig. 1). She was diagnosed with spontaneous rectus sheath hematoma, which was considered to interrupt the blood flow to the right lower limb. Heparin was discontinued, and she received red blood cells transfusions and opted for conservative treatment with bed rest. Despite persistent hematoma, abdominal pain subsided along with gradual improvement in laboratory values, which is hemoglobin 5.8 g/dL to 10.4 g/dL, and limb edema.

## Discussion

In the present case, steroid and anticoagulant therapy used for nephrotic syndrome led to the formation of rectus sheath hematoma. Rectus sheath hematoma is typically associated with a history of trauma. Case reports also indicate cough, childbirth, exercise, and subcutaneous injection as triggers of rectus sheath hematoma, although the cause is unknown in many cases [1]. Rectus sheath hematoma often presents as acute abdomen and is misdiagnosed as appendicitis, hernia, or torsion of the ovarian cystic stem. Computed tomography is the most useful diagnostic modality for rectus sheath hematoma, which is observed as a spindle-shaped mass that is hyperintense compared to the surrounding muscle tissue [3]. Albeit considered a benign presentation, the mortality rate is higher in patients on anticoagulant therapy who tend to harbor larger, clinically more severe hematomas [4]. Many patients can be conservatively treated, whereas invasive treatment, angiography, and embolization or surgical ligation of the bleeding vessels are other reported approaches [4, 5].

The present patient was diagnosed with rectus sheath hematoma during treatment for nephrotic syndrome. Additionally, she harbored several risks factors for vascular failure, including anticoagulant and steroid therapy, older age, and nephrotic syndrome. Massive proteinuria led to the loss of coagulation factors, and it remains possible that steroid and anticoagulant therapy contributed to the effects of endothelial cell damage and arteriosclerosis [6], inducing hematoma formation. In addition, elderly patients are likely more vulnerable due to the progressive weakening of the abdominal wall with age, a condition that can induce severe rectus sheath hematoma [7]. However, she improved without invasive treatment, because of early treatment with blood transfusions and bed rest until the normalization of her hemoglobin level. Fortunately, the hematoma did not become infected, a critical factor in patient outcomes. Rectus sheath hematoma typically presents with abdominal pain, whereas leg edema is a rare presentation. We ruled out other potential etiologies such as deep vein thrombosis, infection, trauma, and exacerbation of medical conditions such as congestive heart failure. One limitation of our study is the inability to establish a clear correlation between vascular failure and treatment outcomes. Due to the poor renal function, the patient could not be evaluated with contrast-enhanced abdominal computed tomography.

The present case illustrates that rectus sheath hematoma should be included in the differential diagnosis of abdominal pain and limb edema in patients on anticoagulant therapy.

## Conclusion

We reported the case of a patient with rectus sheath hematoma following treatment for nephrotic syndrome. Rectus sheath hematoma should be considered in patients with abdominal pain, and timely diagnosis and prompt reperfusion treatment are key components in the successful management of these patients.

## Data Availability

All data generated or analyzed during this study are included in this article. Further enquiries can be directed to the corresponding author.

## References

[1] Hatjipetrou A, Anyfantakis D, Kastanakis M (2015). Rectus sheath hematoma: a review of the literature. Int J Surg.

[2] Takahashi K, Nihei T, Aoki Y, Nakagawa M, Konno N, Munakata A, Okawara K, Kashimura H (2019). Spontaneous rectus sheath hematoma associated with warfarin administration: a case report. J Rural Med.

[3] Jayawardene SA, Goldsmith DJ (2002). Rectus sheath haematomata in patients with renal disease. Nephrol Dial Transplant.

[4] Osinbowale O, Bartholomew JR (2008). Rectus sheath hematoma. Vasc Med (London, England).

[5] Rimola J, Perendreu J, Falco J, Fortuno JR, Massuet A, Branera J (2007). Percutaneous arterial embolization in the management of rectus sheath hematoma. AJR Am J Roentgenol.

[6] Teske JM (1946). Hematoma of the rectus abdominis muscle; report of a case and analysis of 100 cases from the literature. Am J Surg.

[7] Sheth HS, Kumar R, DiNella J, Janov C, Kaldas H, Smith RE (2016). Evaluation of risk factors for rectus sheath hematoma. Clin Appl Thromb/Hemostasis.

